# Is There an Immunohistochemical PD-L1 Cut-Off Point That Serves as a Prognostic Indicator for Large B-Cell Lymphomas? †

**DOI:** 10.3390/diagnostics14111167

**Published:** 2024-05-31

**Authors:** Selcuk Cin, Suat Hilal Aki, Tugrul Elverdi, Deniz Ozmen, Ayse Salihoglu

**Affiliations:** 1 Department of Pathology, Cerrahpasa Faculty of Medicine, Istanbul University-Cerrahpasa, 34098 Istanbul, Turkey; h_aki@yahoo.com; 2 Division of Hematology, Department of Internal Medicine, Cerrahpasa Faculty of Medicine, Istanbul University-Cerrahpasa, 34320 Istanbul, Turkey; streep82@yahoo.com (T.E.); dnzozm@hotmail.com (D.O.); aysesalihoglu@hotmail.com (A.S.)

**Keywords:** immunohistochemical, immunotherapy, cut-off, large B-cell lymphoma, pd-l1, prognosis

## Abstract

The aim of this study is to investigate whether there is a cut-off value for PD-L1 expression in large B-cell lymphomas that predicts prognosis, and to clarify the relationship between PD-L1 expression and histopathological as well as clinical parameters. The study included a total of 130 patients who were diagnosed with large B-cell lymphoma at Istanbul University-Cerrahpasa, Cerrahpasa Faculty of Medicine, Pathology Department. Biopsy samples were assessed using the PD-L1 immunohistochemical antibody (Dako, 22C3 clone). The patients had a mean age of 54 ± 17 years, with a median age of 56 years. No statistically significant difference was observed between the groups in terms of survival when the 30% cut-off value was used. However, a noteworthy discrepancy in survival became apparent when the cut-off point was established at 70%. Among the diffuse large B-cell lymphoma-not otherwise specified (DLBCL-NOS) category, the activated B-cell-like (ABC-like) phenotype showed higher PD-L1 expression compared to the germinal center B-cell-like (GCB-like) phenotype. Immunohistochemical PD-L1 expression emerged as a prognostic factor, particularly significant in the ABC-like phenotype.

## 1. Introduction

Diffuse large B-cell lymphomas (DLBCLs) are the most common aggressive lymphomas among non-Hodgkin’s lymphomas, and account for about 30% of non-Hodgkin’s lymphomas [1,2,3]. Following FDA approval of rituximab in 1997, an anti-CD20 monoclonal antibody, and its inclusion in the standard treatment of DLBCL patients, a cure was attained in around 60% of these cases [4]. Apart from conventional treatments, recently developed immunotherapy can play an important role in the treatment of these patients [4,5,6]. After the discovery of PD-1 in 1992, and research on immune checkpoint regulation has led to the development of various immunotherapy agents, which have found utility in the treatment of various solid cancers. In this context, ’pembrolizumab’ was approved by the FDA for the treatment of classical Hodgkin’s lymphomas in 2016, and primary mediastinal large B-cell lymphoma in 2017 [7,8].

It is understood that the PD-1/PD-L1 pathway regulates the T-cell response to maintain autoimmunity and protect the organism from immune-mediated damage [9,10,11]. Although the PD1/PDL1-2 pathway plays an important role in the development of immune tolerance and the prevention of autoimmune diseases, extended exposure to antigens such as cancer or viral infections leads to the loss of CD8+ cytotoxic T-cell functions (T-cell exhaustion) and subsequent immune system inadequacy [12]. PD-L1, generated by tumor cells, restricts the immune response of the host by binding to PD-1 on the surface of T cells, which inhibits the release of cytokines including interleukin 2 (IL2), tumor necrosis factor alpha (TNFα), and interferon gamma (IFN γ). Consequently, such tumors elude the immune system [12,13]. On the other hand, Treg cells, a subtype of CD4+ T cells, contribute to the establishment of an immunosuppressive environment through the release of inhibitory cytokines such as interleukin 10 (IL-10), transforming growth factor beta by promoting effector T-cell apoptosis via granzyme and perforin, or by leading the depletion of IL2 in the environment via CD25 [14].

In various studies, genetic alterations associated with PD-L1 expression have been identified in around 20% of DLBCL cases [14,15]. This study investigates the potential relationship between immunohistochemical PD-L1 expression, the histopathological and clinical parameters, as well as the prognosis of large B-cell lymphoma (LBCL) cases.

## 2. Materials and Methods

### 2.1. Patients and Clinicopathological Parameters 

After grouping all slides and blocks pertaining to 159 patients sourced from our archives who were diagnosed with LBCL between 2011 and 2019, they were re-evaluated. Among cases with different protocol numbers for the same patient, the most suitable one for the study (considering factors like tissue quantity and presence of artifacts) was chosen. In cases where bone marrow biopsies were available for the same patient, bone marrow biopsy was not preferred due to routine formic acid decalcification before formalin fixation. Twenty-four of the selected cases had insufficient tissue in their blocks, and in the H&E-stained sections of five cases, artifacts such as crushing or freezing were present, resulting in their exclusion from the study. Finally, a total of 130 patients diagnosed with nodal or extranodal LBCL at Istanbul University-Cerrahpasa, Cerrahpasa Medical Faculty Pathology Department, and followed by the Internal Medicine Hematology department, were included in the study. The clinical data for the cases in the study were obtained from pathology reports, patient follow-up files in the hematology department, and the hospital automation system. Survival time was calculated based on the time from first diagnosis date to the death date for deceased patients, and to 1 November 2019 for living patients. 

### 2.2. Histopathological and Immunohistochemical Evaluation 

**Figure 1:**
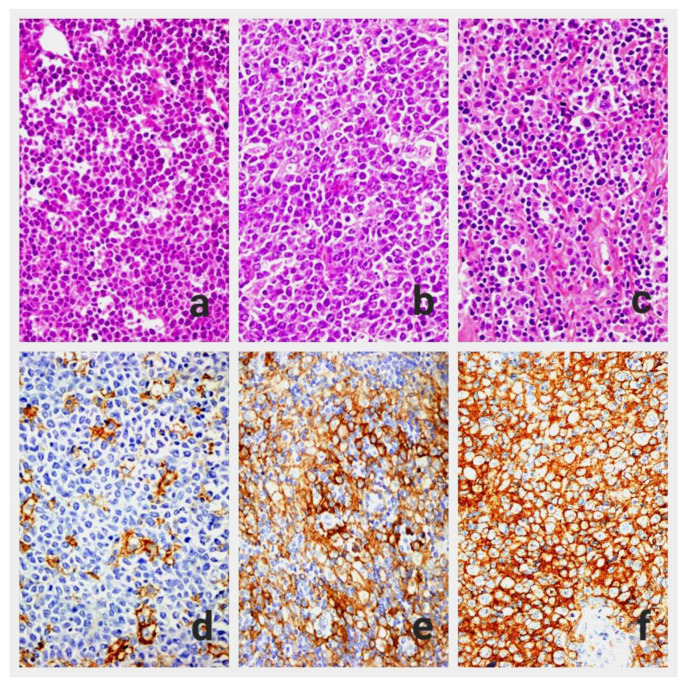
(**a**) DLBCL-NOS, activated B-cell-like, H&E×400 (**b**) DLBCL-NOS, germinal center B-cell-like, H&E×400 (**c**) T-cell-rich B-cell lymphoma, H&E×400, (**d**) immunohistochemical tPD-L1 expression < 30%, (**e**) tPD-L1 expression ≥ 30%, (**f**) tPD-L1 expression almost 100%.

The obtained sections with 2 μm thickness were deparaffinized and rehydrated. Then, the samples were incubated with anti-PD-L1 antibody (for 60 min incubation; 1:50 dilution, monoclonal mouse anti-human PD-L1 clone 22C3, and pharmDx antibody [Dako, Carpinteria, CA, USA]), using a semi-automatic device (Dako Autostainer Link48 and Dako PT link). In the control tissue sections taken from the tonsil, membranous staining was observed in lymphoid cells within the crypt epithelium and, to a lesser extent, in the follicles, which was considered as a positive control. The immunohistochemically PD-L1-stained slides were examined and compared with H&E slides under a light microscope (Figure 1a–c). We identified five hot spot areas where tumor cells showed complete or partial membranous staining at ×400 magnification. These areas were photographed digitally, and the photographs were divided into four equal parts using a digital ruler. The number of tumor cells in each part was counted manually, along with H&E sections, and in some cases with previously applied immunohistochemical slides (CD20, CD3, CD10, BCL2, BCL6). After evaluation of all tumor cells in these regions, regardless of whether they had PD-L1 staining or not, this process was repeated for five different areas. The PD-L1 score for the tumor (tPD-L1) was calculated by determining the ratio of PD-L1 (+) tumor cells to the total number of tumor cells (Figure 1d–f). 

### 2.3. Statistical Analysis

Statistical assessments were conducted using 2 distinct scoring systems. In the first of these, tumor cells were categorized into three groups: those stained below 1%, those stained less than 50% above 1%, and those stained at 50% and above. Apart from this classification, a cut-off value of 30% was established, and statistical analyses were conducted based on this threshold. For an effect size of 0.5, a significance level of 0.05, and a power of 0.80, the minimum total sample size was calculated as 128. In the statistical comparison, the chi-square test, Fisher exact test, and likelihood ratio were used for categorical data. The Fisher exact and likelihood ratio test was used when the expected value of any of the evaluated event tables was <5. Survival was assessed using Kaplan–Meier analysis, and a log-rank (Mantel–Cox) analysis was performed to compare the survival rates among the groups.

For determining statistical significance, a *p*-value of less than 0.05 was considered significant within the 95% confidence interval. Statistical analysis was carried out using the Statistical Package for the Social Sciences (SPSS Inc, Chicago, IL, USA) program, version 21.0. 

## 3. Results

Out of the cases, 70 were male (54%) and 60 were female (46%). The patients’ average age was 54 ± 17 years, with an age range of 18–87 and a median age of 56 years. Among the 130 cases included in our study, 59 (45.4%) were the DLBCL-NOS, ABC-like phenotype; 50 (38.5%) were the DLBCL-NOS, GCB-like phenotype; and 4 (3.1%) were DLBCL-NOS unclassified. Seventeen of cases consisted of special types of large B-cell lymphomas. The clinical characteristics and PD-L1 expressions of the cases are summarized in Table 1.

**Figure 2:**
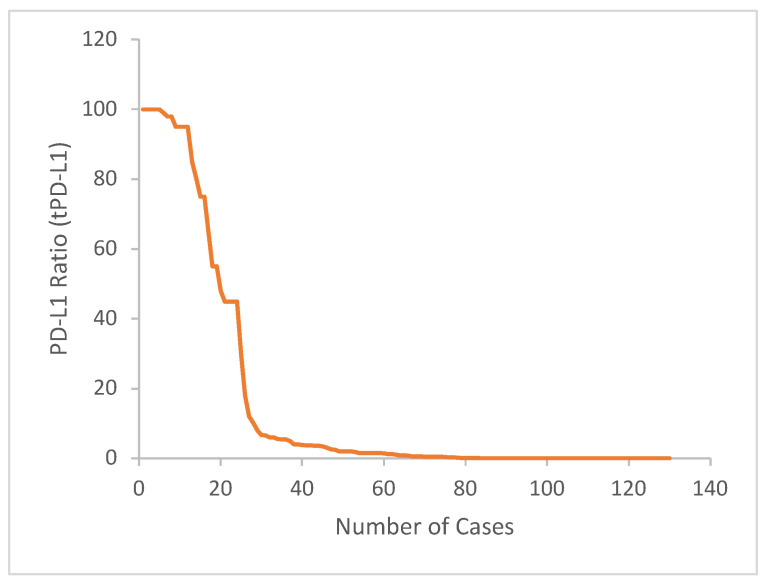
Distribution of cases according to tPD-L1 ratio.

In this study, the tPD-L1 expression was calculated as 19.2%, using the cut-off value of 30%. (Figure 2 shows distribution of cases according to tPD-L1 ratio). The 5-year survival rate was found to be 69.3% for tPD-L1 < 30% cases, and 60.2% for tPD-L1 ≥ 30% cases. The mean survival time was statistically determined as 71.7 ± 3.7 (64.5–78.9) months in the tPD-L1 < 30% group and 50.4 ± 6.7 (37.2–63.5) months in the tPD-L1 ≥ 30% group. There was no significant difference between the groups in terms of survival (log-rank *p*: 0.261). 

There were no significant differences between the groups in terms of gender, age, and B symptoms at 30% cut-off value (*p* > 0.05). At the 30% cut-off point, there was no statistically significant relationship between PD-L1 positivity and clinical parameters such as LDH levels, ECOG performance score, and number of extra nodal involvements (*p* > 0.05). When the cases were evaluated in two groups according to the Ann Arbor stage, a significant difference in PD-L1 expression was observed between the two groups (*p*: 0.032) (Table 2).

**Figure 3:**
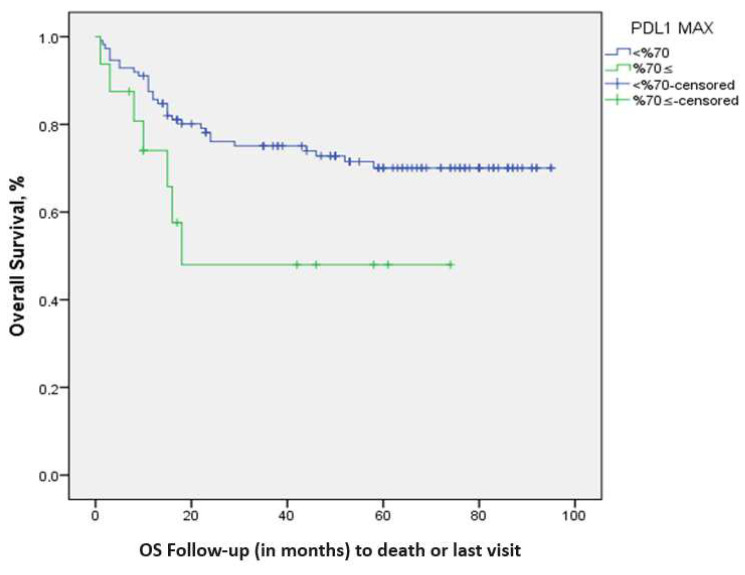
Kaplan–Meier survival analysis for the cut-off value of 70% tPD-L1 staining.

The study investigated the association between PD-L1 expression in the tumor and survival outcomes across different cut-off values: 1%, 5%, 10%, 20%, 30%, 40%, 50%, 60%, 70%, 80%. The survival rate was significantly decreased for cut-off values of 70% and above (*p*: 0.039). The 5-year survival rates were determined as 71.1% in patients with a cut-off value of tPD-L1 expression < 70%, and 48% in patients with a cut-off value of tPD-L1 expression ≥ 70%; the mean survival times were 72.14 ± 3.50 (65.2–79.01) months and 41.2 ± 8.8 (23.8–58.6) months, respectively. A significant difference in terms of survival was observed between the two groups (Figure 3).

There were no significant differences between the groups in terms of gender, age, and B symptoms at the 70% cut-off value (*p* > 0.05) (Table 3).

When the cases were divided into three groups as <1%, 1–49%, and ≥50%, there was no statistically significant difference between tPD-L1 staining and age, gender, LDH level, ECOG performance score, extra nodal involvement, and Ann Arbor stage (Table 4).

The variables included in the univariate analyses were age, B symptom status, LDH level, ECOG performance score, Ann Arbor stage groups, IPI score, and tPD-L1 70% cut-off value. These variables were subjected to Cox regression analysis (Cox regression squared value: 29.488; *p*: 0.001). Notably, tPD-L1 ≥ 70% was identified as a significant prognostic factor (Table 5).

## 4. Discussion

An extensive search through the literature indicates that PD-L1 antibodies have found their place in the treatment of various solid tumor types across different regions, including gastrointestinal tract, breast, and kidney carcinomas [7,16]. Numerous studies have also investigated the PD1/PD-L1 pathway in LBCLs. Upon reviewing the studies, it becomes evident that defining a precise cut-off value is not achievable. In a study conducted by Kwon D. et al. in 2015, a cut-off value of 10% was established for PD-L1 positivity, while in another study by Kiyasu et al., it was set at 30% [6,17]. Moreover, various other cut-off values, such as 5%, 25%, and 50%, have been employed in different studies.

In our study, we found that the proportion of cases exhibiting PD-L1 positivity at a cut-off value of 30% was 19.2%. It is worth noting that this ratio has been reported to vary widely in the literature, ranging from 10.5% to 61.1% [6,17,18,19,20,21,22,23]. In other studies that used the 30% cut-off value, this ratio has been reported as 10.5%, 15.6%, 16.8%, 16%, and 21%, and our findings were consistent with those reported in the literature [6,18,19,22,23]. However, differences in factors such as the PD-L1 antibody clone used, automated counting in either a light microscope or a digital environment, and interobserver variability can be considered as potential reasons for the variations observed. Furthermore, when assessing PD-L1-positive cells within the tumor, various criteria are applied. Some authors consider only membranous staining [24,25], while others deem membranous and/or cytoplasmic staining as positive [6,17,20]. In certain studies, staining intensity is used for evaluation [21,22,23], whereas in others, including our study, the focus is solely on whether tumor cells are stained or not [18,24,25]. In LBCL, cells such as histiocytes may sometimes exhibit PD-L1 staining, leading to potential misinterpretations. As a result, some researchers have embraced the utilization of ‘combined PD-L1 positivity’ (CPS). On the other hand, dual staining with PD-L1 and PAX-5 antibodies, serving as a B-cell indicator together, as demonstrated in Kiyasu et al.‘s study, can help reduce false positive results [6]. Nevertheless, in routine practice, assessing dual staining requires considerations of cost effectiveness and benefits. Due to these technical differences, reaching a consensus on PD-L1 expression appears challenging. However, as more studies are conducted, it may become possible to obtain more accurate results by standardizing the methodologies.

Varying results have been reported in studies investigating the relationship between tPD-L1 positivity and survival in large B-cell lymphomas. While some studies have linked tPD-L1 positivity to a poor prognosis and low overall survival, others have found no such correlation. In 2015, Kwon D. and colleagues conducted a study with 126 patients using a cut-off value of 10%. They observed that in patients treated with R-CHOP, PD-L1 expression in the tumor had no significant prognostic impact [17]. On the other hand, Kiyasu and colleagues, in a study involving 1253 patients with a cut-off value of 30%, demonstrated that tPD-L1 expression served as an independent prognostic factor for overall survival [6]. In another study with 76 patients, Fang X. and colleagues noted an association between PD-L1 expression and a poor prognosis, but it was not deemed an independent prognostic factor [26]. In our study, we observed no significant difference in 3-year and 5-year overall survival rates between the groups using the 30% cut-off value. However, we investigated the relationship between PD-L1 expression in the tumor and survival across a range of cut-off values, including 1%, 5%, 10%, 20%, 30%, 40%, 50%, 60%, 70%, and 80%. Since all the cases were diagnosed with LBCL in our study and there was no control group to compare, we could not use the ROC analysis to determine the cut-off value. Among the binary groups formed using these cut-off values, a significant difference in survival emerged when a 70% threshold was used. In Cox regression analysis, tPD-L1 levels of 70% or higher were found to pose a 2.65 times higher risk in terms of survival. This led to the conclusion that PD-L1 expression is correlated with poor prognosis and serves as an independent prognostic factor. While there was no observed relationship between survival and tPD-L1 staining at a cut-off value of 30%, the identification of such an association at a higher cut-off value underscores the need for more comprehensive studies on this matter.

In our study, we conducted analyses not only using a single cut-off value, but also employing different grouping methods. Survival analyses, performed in three groups (<1%, 1–50%, 50% and above), did not reveal a significant difference between these groups.

In this study, a notable association was observed between the Ann Arbor stage, a critical determinant of patient prognosis, and tPD-L1 positivity for the 30% threshold. When patients with stages 3 and 4 were grouped together and those with stages 1 and 2 were grouped separately, significant tPD-L1 staining was observed in the former group. There are many publications that do not show such a correlation, but there are also studies supporting this finding [6,17,18,20,22,23,24,25,26,27]. A clear consensus is challenging to establish in studies, except for age and LDH-level parameters of the IPI score. Apart from variations in tPD-L1 evaluation methods, factors such as tumor heterogeneity, time to diagnosis, and the utilization of appropriate imaging methods to assess extranodal involvement may have contributed to these differing results.

In our study, apart from DLBCL-NOS, there were 17 cases found that encompass other large cell lymphoma subtypes, which may have caused heterogeneity in the cases. However, when we separately evaluated DLBCL-NOS cases (113 patients) and conducted our statistical analyses, we obtained similar results. The DLBCL-NOS, ABC-like phenotype exhibited a higher tPD-L1 expression rate compared to the GCB-like phenotype, and these cases had lower survival rates. This particular pattern appears consistently across numerous studies in the literature [6,17,18,20,22,23,25,27]. While our study showed no survival difference at the 30% cut-off value, a significant survival discrepancy was evident between these two groups at the 60% cut-off value. This discrepancy became even more pronounced at the 70% cut-off value (*p*: 0.002). It is worth noting that there is a significant survival difference, particularly at a lower tPD-L1 cut-off level (60%, not 70%), when compared to all cases of large B-cell lymphomas, specifically in the DLBCL-NOS group.

## 5. Limitations

The lack of a control group and a cut-off value to compare immunohistochemical PD-L1 positivity makes it difficult to compare both of the cases within this study as well as our findings with other research in the literature. Additionally, assessing the expression of PD-L1 can be difficult in some cases due to the potential expression of PD-L1 by cells other than tumor cells.

In our cohort, there are 121 patients who were under hematological follow-up, received R-CHOP therapy, and had complete data available. However, there were variations found among patients in terms of dose adjustments and delays, salvage regimens, and transplantation. Although no statistically significant difference was observed between treatment responses and PD-L1 expression, investigating whether different treatment regimens have any effect on PD-L1 expression remains a subject of inquiry.

## 6. Conclusions

In our study, we observed a significant difference in tPD-L1 expression and survival at the cut-off value of 70%. While there are varying results in the literature concerning the relationship between PD-L1 expression and survival rates in LBCLs, conducting multicenter studies with larger sample sizes and different subtypes can provide valuable insights. This may shed light on the mechanism of PD-L1 expression in the tumor and whether patients, especially those in treatment-resistant or relapsing disease groups, can benefit from anti-PD-L1 therapy.

## Figures and Tables

**Table 1:** Clinicopathologic characteristics of patients *.

		Number	Ratio
Sex	Male	70	53.8
	Female	60	46.2
Age	<60 years	75	57.7
	≥60 years	55	42.3
B Symptoms	Positive	89	69
	Negative	40	31
Survey	Deceased	38	29.2
	Alive	92	70.8
LDH Level	Normal	53	41.1
	Elevated	76	58.9
ECOG Performance Score	<2	105	81.4
	≥2	24	18.6
Extranodal Involvement	<2	63	48.8
	≥2	66	51.2
Ann Arbor Stage	Stage 1	21	16.3
	Stage 2	29	22.5
	Stage 3	19	14.7
	Stage 4	60	46.5
	Stages 1 and 2	50	38.8
	Stages 3 and 4	79	61.2
IPI Score	0	15	11.6
	1	27	20.9
	2	23	17.8
	3	33	25.6
	4	26	20.2
	5	5	3.9
R-CHOP Treatment ^#^	RCHOP (+)	121	0.8
	RCHOP (−)	1	99.2
PD-L1 Positivity	<1%	67	51.5%
	1–49%	44	33.8%
	>50%	19	14.6%

* Some of the clinical information of one patient could not be accessed. ^#^ Treatment information of eight patients could not be obtained.

**Table 2:** Relationship between PD-L1 expression and clinical parameters for the cut-off value of 30% *.

		tPD-L1 Staining Percentage					
		<%30		≥%30			
		*n*	%	*n*	%		*p*
Sex	Male	54	77.1	16	22.9		0.257
	Female	51	85.0	9	15.0		
Age	<60 years	63	84.0	12	16.0		0.275
	≥60 years	42	76.4	13	23.6		
B Symptoms	+	74	83.1	15	16.9		0.279
	−	30	75.0	10	25.0		
LDH Levels	Normal	46	86.8	7	13.2	0.188	
	Elevated	59	77.6	17	22.4		
ECOG Performance Score	<2	86	81.9	19	18.1	0.567	
	≥2	18	75.0	6	25.0		
Exranodal Involvement	<2	52	82.5	11	17.5	0.590	
	≥2	52	78.8	14	21.2		
Ann Arbor Stage	Stages 1 and 2	45	90.0	5	10.0	0.032	
	Stages 3 and 4	59	74.7	20	25.3		

* Some of the clinical information of one patient could not be accessed.

**Table 3:** Relationship between PD-L1 expression and clinical parameters for the cut-off value of 70% *.

		tPD-L1 Staining Percentage					
		<%70		≥%70			
		*n*	%	*n*	%		*p*
Sex	Male	60	85.7	10	14.3		0.458
	Female	54	90.0	6	10.0		
Age	<60 years	65	86.7	10	13.3		0.678
	≥60 years	49	89.1	6	10.9		
LDH Levels	Normal	48	90.6	5	9.4	0.516	
	Elevated	66	86.8	10	13.2		
ECOG Performance Score	<2	93	88.6	12	11.4	0.497	
	≥2	20	83.3	4	16.7		
Exranodal Involvement	<2	56	88.9	7	11.1	0.664	
	≥2	57	86.4	9	13.6		
Ann Arbor Stage	Stages 1 and 2	46	92.0	4	8.0	0.227	
	Stages 3 and 4	67	84.8	12	15.2		

* Some of the clinical information of one patient could not be accessed.

**Table 4:** Relationship between PD-L1 expression and clinical parameters for three groups *.

		tPD-L1 Staining Percentage						
		<%1		%1–49		≥%50		
		*n*	%	*n*	%	*n*	%	*p*
Sex	Male	37	52.9	20	28.6	13	18.6	0.232
	Female	30	50.0	24	40.0	6	10.0	
Age	<60 years	44	58.7	20	26.7	11	14.7	0.108
	≥60 years	23	41.8	24	43.6	8	14.5	
LDH Levels	Normal	30	56.6	17	32.1	6	11.3	0.627
	Elevated	37	48.7	27	35.5	12	15.8	
ECOG Performance Score	<2	54	51.4	37	35.2	14	13.3	0.499
	≥2	13	54.2	6	25.0	5	20.8	
Extranodal Involvement	<2	34	54.0	21	33.3	8	12.7	0.802
	≥2	33	50.0	22	33.3	11	16.7	
Ann Arbor Stage	Stage 1	15	71.4	5	23.8	1	4.8	0.199
	Stage 2	17	58.6	9	31.0	3	10.3	
	Stage 3	6	31.6	8	42.1	5	26.3	
	Stage 4	29	48.3	21	35.0	10	16.7	

* Some of the clinical information of one patient could not be accessed.

**Table 5:** Cox regression analysis.

		HR *	%95 CI ^#^		*p*
Age groups	<60 vs. ≥60	2.267	2.267	1.100	0.027
B Symptoms	Absent vs. exist	1.593	0.749	3.390	0.227
LDH Levels	Normal vs. elevated	0.545	0.178	1.673	0.289
ECOG Performance Score	<2 vs. ≥2	1.427	0.622	3.277	0.401
Ann Arbor Stage	Stage I/II vs. III/IV	0.869	0.287	2.628	0.804
IPI Score	0/1/2 vs. 3/4/5	4.658	1.073	20.211	0.040
tPD-L1 Staining Percentage	<%70 vs. ≥70	2.646	1.049	6.675	0.039

* Hazard ratio; ^#^ confidence interval.

## Data Availability

The original contributions presented in the study are included in the article, further inquiries can be directed to the corresponding author.
